# SET Kinetics of Ag/HfO_2_-Based Diffusive Memristors under Various Counter-Electrode Materials

**DOI:** 10.3390/mi14030571

**Published:** 2023-02-27

**Authors:** Solomon Amsalu Chekol, Richard Nacke, Stephan Aussen, Susanne Hoffmann-Eifert

**Affiliations:** 1 Peter Grünberg Institute (PGI 7 and 10) and JARA-FIT, Forschungszentrum Jülich GmbH, Wilhelm-Johnen-Straße, 52428 Jülich, Germany; 2 Faculty of Georesources and Materials Engineering, RWTH Aachen University, Intzestraße 1, 52072 Aachen, Germany; 3 Faculty of Mathematics, Computer Science and Natural Sciences, RWTH Aachen University, Templergraben 59, 52062 Aachen, Germany

**Keywords:** diffusive memristor, volatile switches, kinetics, electrochemical metallization, electrocatalytic

## Abstract

The counter-electrode (CE) material in electrochemical metallization memory (ECM) cells plays a crucial role in the switching process by affecting the reactions at the CE/electrolyte interface. This is due to the different electrocatalytic activity of the CE material towards reduction–oxidation reactions, which determines the metal ion concentration in the electrolyte and ultimately impacts the switching kinetics. In this study, the focus is laid on Pt, TiN, and W, which are relevant in standard chip technology. For these, the influence of CE metal on the switching kinetics of Ag/HfO_2_-based volatile ECM cells is investigated. Rectangular voltage pulses of different amplitudes were applied, and the SET times were analyzed from the transient curves. The results show that CE material has a significant effect on the SET kinetics, with differences being observed depending on the voltage regime. The formation of interfacial oxides at the CE/electrolyte interface, particularly for non-noble metals, is also discussed in relation to the findings. Overall, this work highlights the important role of the CE material in the switching process of Ag/HfO_2_-based diffusive memristors and the importance of considering interfacial oxide formation in the design of these devices.

## 1. Introduction

Brain-inspired neuromorphic computing aims to create computing systems that are more efficient, adaptable, and intelligent than traditional computers by using novel architectures and algorithms [1,2,3]. One major challenge is the need for emerging memories that are scalable to large arrays and consume low power to support the requirements of these systems. One such emerging memory technology is redox-based resistive random access memory (ReRAM), owing to its simple structure, non-volatility, low power consumption, and suitability for high-density integration [4]. One type of ReRAM is the electrochemical metallization memory (ECM), also known as conductive bridge RAM, which relies on the formation and rupture of a conducting metal bridging filament between two electrodes to change the resistance of the cell [5,6,7,8]. This conductive filament is composed of active metals such as Ag or Cu. ECM cells mainly consist of a stack of three layers: the counter electrode (CE), the electrolyte (switching) layer, and the chemically active electrode (AE). The CE and AE serve as the two electrodes, and the electrolyte layer provides the ionic-electronic conductivity necessary for the formation of the metallic filament. In standard non-volatile ECM, the programmed data are retained for up to 10 years.

In addition, there are also volatile ECM cells, which lose the programmed low resistance state (LRS) when biasing is stopped [9,10,11]. This is achieved through the formation of a thin (a few nm in diameter) and unstable filament, which spontaneously ruptures within a certain amount of time once the voltage bias is removed, causing the cell to restore the initial high resistance state [12]. Volatile ECM cells, hereinafter referred to as diffusive memristors (DMs), are useful for a variety of applications in data storage and neuromorphic computing areas, such as selector [11], artificial neuron [13], true random number generator [14,15], and short-term synapse [16,17].

Both non-volatile ECMs and DMs have a similar operating mechanism and are based on the formation and rupture of a conducting metal bridge between the two electrodes [6,18]. When a positive voltage is applied to the AE, while the CE is grounded, the active metal is anodically dissolved, and metal ions drift through the electrolyte toward the CE. Depending on the type of electrolyte and device stack, the diffused ion will be electrochemically reduced back to a metallic atom, either inside the electrolyte or at the CE surface [19]. With a continuous redox reaction, the metallic filament begins to grow and finally bridges the gap between the two electrodes.

The CE plays a crucial role in the switching process as it provides the electrons for the reduction reaction, which in turn influences the switching kinetics of the ECM cell. Luebben et al. [20] showed differences in the SET kinetics of non-volatile Ag/SiO_2_-based ECM devices based on a variation of the CE material between Pt, TiN, IrO, and Ru. The authors correlated the observed results to the electrocatalytic activity of the CE material, which affects the Ag ion concentration in the electrolyte and, hence, the switching speed. In particular, electrodes with higher electrocatalytic activity allow for faster switching [21].

For a device to be commercially viable, it must operate at a reasonably high switching speed. This means that the device must be able to quickly and efficiently switch between different states or modes of operation. As discussed, one important factor for the switching speed is the choice of the CE. Therefore, a physical understanding of the impact of the CE on switching kinetics will allow researchers and engineers to optimize the CE design and improve the device’s overall performance.

While the SET kinetics of non-volatile ECM cells has been intensively studied [20,22,23,24,25], here, we look at how the switching kinetics and device performance of DMs are affected by different CE materials. For this study, inert Pt metal and two non-noble metals widely used in semiconductor technology, namely, W and TiN, are used as the CE in Ag/HfO_2_/CE-based DM cells. This study has two main goals: (i) understanding the influence of the CE material on the switching process of DMs, and (ii) device engineering and optimization towards industrially used and CMOS-compatible materials such as TiN and W.

## 2. Experimental Section

The DM devices were fabricated as micrometer crossbar junctions on a Si wafer with a 430 nm SiO_2_ layer. A 5 nm thick Ti film from sputtering serves as an adhesion layer between the substrate and CE. For the DM devices with Pt CE, a sputtered Pt film of 30 nm in thickness was used. For the DMs with TiN and W CEs, the bottom electrode was built up from a stack of 10 nm Pt and 20 nm of the respective CE metal. All layers are deposited by the sputtering technique. The 10 nm Pt layer was added to reduce the final resistance of the crossbar-shaped bottom electrode. Next, the bottom electrodes were lithographically patterned and structured by reactive ion beam etching (RIBE). Afterwards, a 3 nm thick HfO_2_ insulating film was grown on top of the electrodes at 250 °C using a plasma-enhanced atomic layer deposition (PE-ALD) process from tetrakis (ethymethylamino) hafnium (TEMA-Hf) and remote oxygen plasma [26]. The PE-ALD-grown HfO_2_ film has a low defect density, enabling the fabrication of DM devices with nanometer-thin switching layers. Afterwards, the top electrode areas were patterned by photolithography. Subsequently, the microstructured top electrodes were obtained from sputter deposition of 20 nm thick Ag active and 20 nm thick Pt capping layers, followed by a conventional liftoff process. Details on the sputtering parameters for the bottom and top electrodes can be found in Appendix A (Table A1). Finally, the bottom electrode contacts are opened using photolithography and RIBE, resulting in the completed crossbar structures with an area of (8.0 ± 0.2) μm^2^.

The CE film quality was analyzed using atomic force microscopy (AFM) and X-ray photoelectron spectroscopy (XPS) on separate control samples. For XPS measurements, a VersaProbe 5000 manufactured by Physical Electronics (Chanhassen, MN, USA) was used. Monochromatic X-rays are generated by an Al Kα source with an excitation energy of 1486.6 eV. The low-power mode with 25 W and an X-ray spot diameter of about 100 µm was used. To compensate for charging effects, electron neutralization was performed with a neutralizer emission of 20 µA current and a neutralizer bias of 1.37 eV. Survey scans were performed with 187 eV pass energy and core-level scans with 23.5 eV. The binding energies were corrected for electrical charge effects by referencing the C 1 s peak, which was set to 285 eV. Regarding the XPS core-level analyses, a Shirley function for background subtraction was used followed by data fitting with Gaussian–Lorentzian profiles for oxide components and Lorentzian-asymmetric profiles for metallic components. AFM allows for the measurement of surface roughness and other topographical characteristics, while XPS is used to analyze the chemical composition and valence state of the CE components and to identify additional surface or interface layers arising from the processing.

Voltage sweep characterizations were carried out in a probe station using an Agilent B1500A semiconductor device parameter analyzer with high-resolution source and measurement units. The voltage sweep measurements were performed by applying a positive voltage to the Pt/Ag top electrode while the bottom electrode is grounded. Transient voltage pulse experiments were performed using a Keithley 4200A SCS Semiconductor Characterization System equipped with four 4225 PMUs and an integrated oscilloscope card with a bandwidth of 1 GHz. For the pulse experiments, the bottom electrode was grounded, and the voltage pulse signal was applied to the Pt/Ag top electrode, as well. The applied voltage was measured on Channel 1 of the oscilloscope (internal impedance 50 Ω), and the output current was calculated from the post-device under test (DUT) signal measured on Channel 2 (also with an internal impedance of 50 Ω). A custom-built tungsten probe tip with a 100 kΩ surface-mounted device (SMD) resistor was used during the measurement to protect the device from uncontrolled current overshoots.

## 3. Results and Discussion

**Figure 1:**
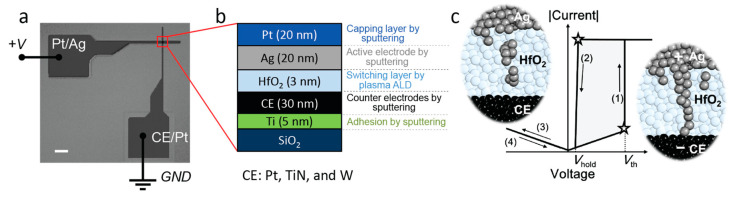
(**a**) Optical micrograph of a fabricated crossbar structure of an Ag/HfO_2_/CE device with CE = Pt. Scale bar 10 μm. (**b**) Schematic of the fabricated device stack at the crossing point of the top and bottom electrodes. (**c**) Representation of the switching characteristics and mechanism of a diffusive memristor.

Figure 1a shows an optical microscopy image of a fabricated crossbar device containing the Pt/Ag top and CE/Pt bottom electrodes. A cross-sectional stack of the active region (inside the red box in Figure 1a) is schematically shown in Figure 1b. 

### 3.1. Surface Oxidation of Counter-Electrode Layer

TiN and W are non-noble metals, which means they are prone to surface oxidation when exposed to an oxygen environment [27,28]. This is because they are not as chemically stable as noble metals, such as Au and Pt. Formation of an oxide surface layer is unavoidable in the case of TiN and W bottom electrodes, as these are exposed to the oxygen atmosphere during the vacuum breaking for the patterning and structuring process, as well as to oxygen radicals during the oxygen plasma step of the HfO_2_ ALD process. In situ transfer of the samples between the various deposition tools could help to minimize surface oxidation. However, this requires modification of the fabrication flow, and, in addition, oxidation at the HfO_2_/W (or HfO_2_/TiN) interface cannot be avoided in any case. Therefore, interfacial metal oxide layers should be considered when discussing the performance of ECM devices made from an oxide electrolyte layer and a non-noble CE. 

**Figure 2:**
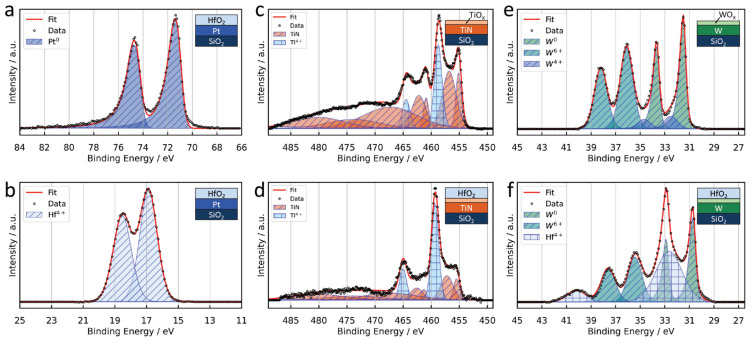
XPS analyses performed on different counter-electrode metal surfaces and interfaces with an HfO_2_ layer on top. (**a**) Pt 4f and (**b**) Hf 4f core-level spectra for a Pt film with a 3 nm HfO_2_ layer on top. (**c**) Ti 4f core-level spectra for a TiN film after exposure to air. (**d**) Ti 4f core-level spectra for a TiN film after deposition of 3 nm HfO_2_. TiO_2_-type layers are found on the TiN surface after exposure to air as well as at the HfO_2_/TiN interface. (**e**) W 4f core-level spectra for a W film after exposure to air. (**f**) W 4f core-level spectra for a W film after deposition of 3 nm HfO_2_. The surface layer of the W metal film consists of WO_3_ and WO_2_. The tungsten oxide interface is more difficult to analyze due to the overlapping signal from HfO_2_. All measurements were done under a take-off angle of 45°.

To better understand the formation and properties of the interfacial oxide on the surface of the CE materials used in this work (Pt, TiN, and W), we conducted XPS analysis on samples of these materials with and without the HfO_2_ layer. XPS is a surface-sensitive analytical technique that allows for the identification and quantification of chemical elements in a sample, as well as the determination of their local valence states. This helps to determine and characterize surface and interfacial oxide layers and discuss the potential impact on the device’s performance. The results from the XPS analysis are shown in Figure 2. First, we investigated the layer stack of 3 nm HfO_2_/30 nm Pt/SiO_2_/Si, where the HfO_2_ film is grown by the PE-ALD process. The analysis of the Pt 4f core-level signal in Figure 2a shows that the Pt layer up to the HfO_2_/Pt interface is pure Pt metal, as only the Pt^0^ doublet with Pt 4f_7/2_ at 71.0 eV is present without any indications about additional valence states. This finding is consistent with the general oxidation behavior of Pt. Moreover, the Hf 4f core-level signal in Figure 2b can be successfully simulated only by the Hf^4+^ doublet peak with a binding energy of 16.9 eV for the Hf 4f_7/2_, indicating that the chemical composition of the whole layer is pure HfO_2_. Therefore, the structure of the DM devices with Pt CE is accurately described by Ag/HfO_2_/Pt.

For the TiN and W CEs, we performed XPS analysis on two different samples for each material: (i) the pure TiN and W surfaces after they were exposed to air, and (ii) the HfO_2_/TiN and HfO_2_/W interfaces, which were obtained after the HfO_2_ layer was grown by PE-ALD on the TiN and W CEs, respectively. This allowed us to compare the properties of the pure TiN and W surfaces to those of the HfO_2_/TiN and HfO_2_/W interfaces and assess the impact of the HfO_2_ PE-ALD process on the formation and properties of the interface. Figure 2c shows the Ti 4f core-level spectrum for the TiN film after it has been exposed to air. Additionally to the peaks attributed to TiN, the spectrum clearly shows Ti^4+^ contribution with the Ti 2p_3/2_ at 459.3 eV. This is attributed to a TiO_2_ surface layer that has been formed when the TiN film was exposed to air. Contributions from Ti^3+^ and Ti^2+^ have been neglected as the corresponding peaks overlap with contributions from TiN and could not be distinguished. However, it is expected that the TiO_x_ is not stoichiometric TiO_2_, but contributions from Ti^3+^ and Ti^2+^ are present. For the HfO_2_/TiN sample (Figure 2d) the intensity of the Ti^4+^ doublet is further increased compared to the TiN signal. This indicates that the TiO_2_ interface layer formed after HfO_2_ PE-ALD onto the TiN film has an increased thickness compared to the surface oxide formed when the TiN film was exposed to air. 

Similarly, Figure 2e shows the W 4f core-level spectrum for the plane W CE after exposure to air. Besides the W^0^ peaks of the tungsten metal with the W 4f_7/2_ at 31.5 eV, additional components appear, which are identified as originating from W^4+^ with a binding energy of 32.5 eV for the W 4f_7/2_ and 36.1 eV for W^6+^. From this, it is concluded to be a WO_3_ surface layer formed on the W metal film after exposure to air. The appearance of a WO_2_ component can hint towards an oxygen gradient when traversing from the WO_3_ surface layer to the W film. The W 4f core-level spectrum for the HfO_2_/W sample (Figure 2f) also shows peaks from W^6+^, which are attributed to a tungsten oxide interface layer formed between the W metal CE and the HfO_2_ electrolyte layer. However, here, it is difficult to accurately fit the WO_2_ peaks due to their overlap with the Hf 5p core level of the HfO_2_ layer. 

**Figure A1:**
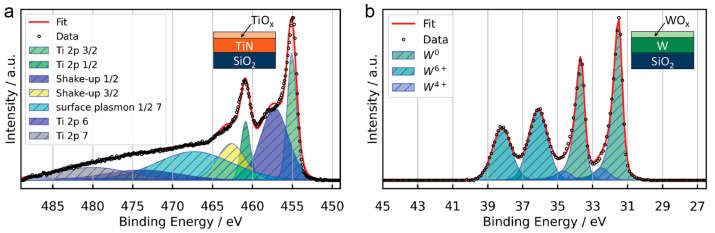
(**a**) XPS analysis of an in situ sputter-cleaned TiN counter-electrode surface showing a pure TiN surface. The sample was measured under a take-off angle of 45°. (**b**) XPS analysis of a pure W film under a take-off angle of 15° targeting the bulk of the film. Both measurements confirmed that the native oxide is formed only at the surface of the counter electrode.

It is important to mention that the native surface oxides that are formed after exposure of the CEs to air are only a few nm in thickness. This has been confirmed by performing XPS on two samples: (i) a TiN surface that has been sputter-cleaned in situ and (ii) an oxidized W surface analyzed with a take-off angle of θ = 15° to target the bulk of the film. The results of these experiments are provided in Appendix A, in Figure A1. In these measurements, metallic phases of TiN and W are more pronounced, indicating that the native oxide layer is only present at the surface of the CEs and does not extend into the bulk of the material. The thickness of the native oxide layer was quantified using x-ray reflectivity (XRR) measurements, which provided values in the range of 2 to 3 nm, consistent with the range of typical native oxide thicknesses [29].

Summarizing, the structures of the DM device stacks can be described as Ag/HfO_2_/Pt, Ag/HfO_2_/TiO_x_/TiN, and Ag/HfO_2_/WO_x_/W.

### 3.2. Forming and Threshold Switching Characteristics 

**Figure 3:**
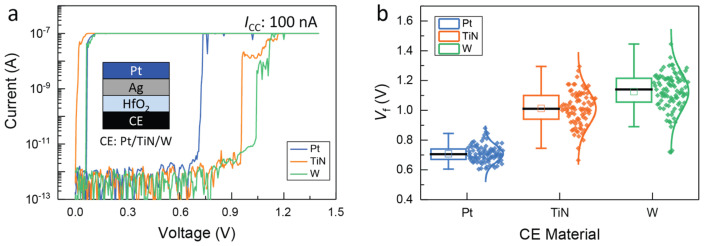
(**a**) Representative *I-V* curves of the forming of Ag/3 nm HfO_2_/CE devices for the three different CE materials Pt, TiN, and W. (**b**) Box plot displaying forming voltage (*V*_f_) collected from 96 cells for Pt, TiN, and W devices. A compliance current (*I*_CC_) of 100 nA and a sweep rate of 6.25 mV/s were used for the measurements.

As-fabricated devices often require a higher voltage to initialize switching during the first cycle, which is known as the forming process. During this process, Ag ions are loaded into the electrolyte layer and form a conductive metal filament. During the subsequent cycles, the switching takes place by rupture and reformation of the conductive filament, thus requiring much less voltage for the SET event. Representative forming curves measured for the three different DM cells of this work are shown in Figure 3a. All three types of devices exhibit a very low initial leakage current in the sub-pico ampere range. When a higher voltage is applied, the cells undergo a sudden transition from the high resistive state (HRS) to the low resistive state (LRS) at a specific forming voltage (*V*_f_). Figure 3b shows the statistical distribution of *V*_f_ for each device type based on the measurements of 96 cells. For the various CEs, *V*_f_ increases in the order: Pt < TiN < W. Additionally, the distribution of the *V*_f_ follows this same trend. This indicates that the Ag/HfO_2_/Pt device requires lower *V*_f_ than the Ag/HfO_2_ devices with TiN and W CE, and the spread of *V*_f_ is narrower for the devices with Pt CE compared to the ones with TiN and W electrodes. This observation can be explained if the formation of an interfacial oxide layer is considered as described in the previous paragraph and confirmed by XPS and XRR analysis data. In particular, for the electroforming process, the total thickness of the insulating electrolyte layer is of superior importance since it determines the electric field applied to the dielectric. Therefore, a thicker oxide layer will require a higher voltage for the conductive filament to form [30,31].

**Figure 4:**
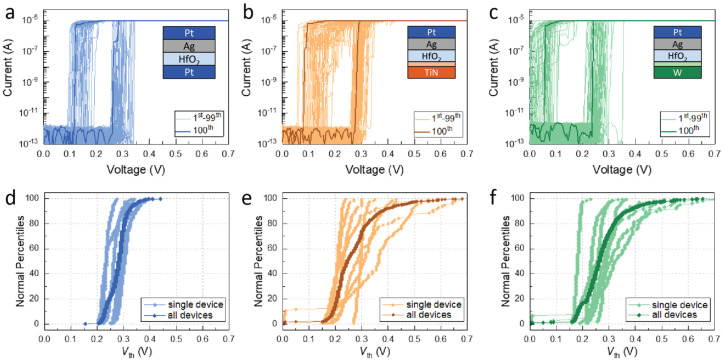
Typical *I-V* curves for Ag/3 nm HfO_2_/CE devices with different counter-electrode materials of (**a**) Pt, (**b**) TiN, and (**c**) W. Statistics of the threshold voltage (*V*_th_) collected from 8 devices and 100 cycles each for (**d**) Pt, (**e**) TiN, and (**f**) W CE. The TiO_x_ and WO_x_ interfacial oxides are shown with light orange and light green colors, respectively. A sweep rate of 6.25 mV/s was used for the measurements.

In contrast to the forming process, the threshold switching behavior of the Pt, TiN, and W CE devices is quite similar, as shown in Figure 4a–c. The diagrams show that all three devices switch to the LRS at a threshold voltage (*V*_th_) of approximately 0.2–0.3 V, in an abrupt, volatile, and reproducible manner. This indicates that the devices with Pt, TiN, and W CE have similar switching characteristics once the forming process has been completed. However, the cycle-to-cycle (c2c) and device-to-device (d2d) variations of the *V*_th_ values differ for the three types of DMs. *V*_th_ values collected from 8 random devices switched for 100 cycles each are plotted for each device in Figure 4d–f. Devices with Pt CE have the least deviation in both c2c and d2d, with only a small spread in the switching behavior. In contrast, those with TiN CE exhibit moderate c2c variation for most devices, but a few outliers display much higher variation. Devices with W CE also show stable switching behavior, but the spread in the c2c and d2d variations is larger than for the other two types of devices.

The difference in the variation can be attributed to an inhomogeneity of the switching layer in devices with TiN and W CE, which include a metal oxide interfacial layer in addition to the 3 nm HfO_2_ electrolyte layer. In addition, the increased total thickness of the insulating layer comes along with an increased *V*_f_ and higher variation in the switching behavior.

**Figure 5:**
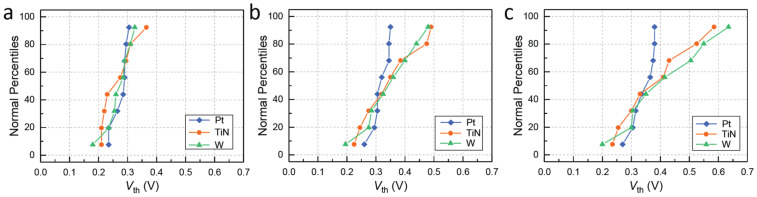
Distribution of the device-to-device variations in the threshold voltage (*V*_th_) for the three counter-electrode devices at the (**a**) 50th percentile (mean), (**b**) 90th percentile, and (**c**) 97th percentile.

When examining the d2d variation in *V*_th_, the mean *V*_th_ is more or less similar for devices with Pt, TiN, and W CE, as shown in Figure 5a. However, when looking at the data from the 90th (Figure 5b) and 97th (Figure 5c) percentiles of the distributions, differences between the three device types become apparent. Devices with Pt CE show a tight distribution almost independent of the selected percentile, while the distributions of the devices with TiN and W CE become less steep and start to deviate more from the mean. Therefore, the effect of the CE material on the switching performance of the DMs derived from continuous *I-V* loops mainly manifests in larger deviations and increased contributions from outliers.

### 3.3. SET Kinetics

**Figure 6:**
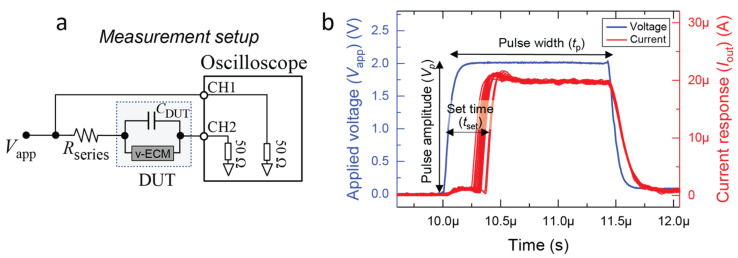
(**a**) The measurement setup used to perform all transient pulsed measurements in this work. (**b**) Typical transient current response of an Ag/HfO_2_/Pt device under a 2.00 V/1.5 μs programming pulse. A 100 kΩ series resistor (*R*_series_) was used to limit the maximum current.

Understanding the switching kinetics of ECMs and how different CE materials affect the kinetics is essential for improving the performance of emerging memristor-based technologies. To that end, the SET time (*t*_set_) of all three types of devices was analyzed by using programming pulses with varying pulse amplitude (*V*_p_) and width (*t*_p_), ranging from 0.30 to 2.20 V and 1 μs to 1 s, respectively. The data are collected from multiple pulse widths to access the different times across the different voltages, as the minimum rise time is limited for a given *t*_p_. However, *t*_p_ does not have any influence on the SET kinetics [32]. A minimum rise time of 30 ns was used for the shortest *t*_p_. Voltage steps of 0.05 V and time steps of one order of magnitude were used in the analysis. *t*_set_ is the amount of time it takes for the device to switch to the LRS when a voltage pulse is applied. The measurement setup used in this work is depicted in Figure 6a in the form of a circuitry schematic. Figure 6b shows a typical current response of an Ag/HfO_2_/Pt device measured using a 2.00 V/1.5 μs programming pulse. 

**Figure 7:**
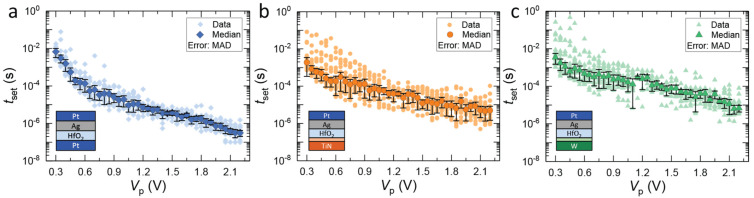
SET switching kinetics of Ag/HfO_2_/CE DM cells for the three counter electrodes of (**a**) Pt, (**b**) TiN, and (**c**) W. The median values and median absolute deviations (MAD) of the experimental data are displayed using color-filled symbols and vertical lines, respectively. The TiO_x_ and WO_x_ interfacial oxides are shown with light orange and light green colors, respectively. A 100 kΩ series resistor (*R*_series_) was used to limit the maximum current in all measurements.

**Figure 8:**
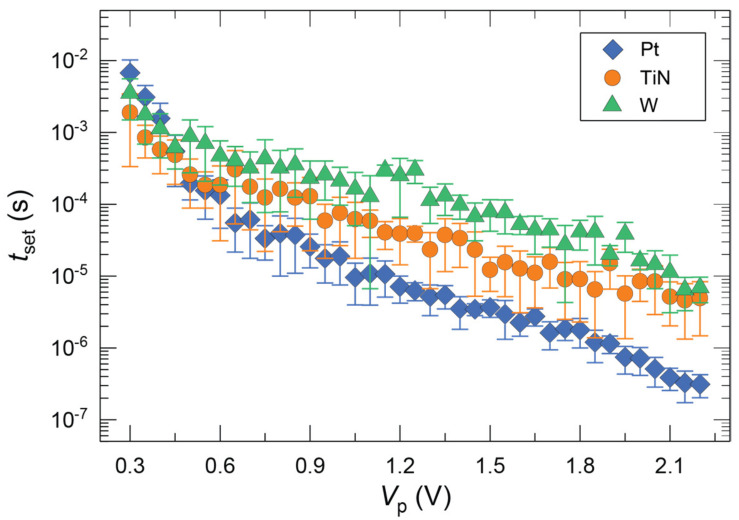
A SET kinetics plot of the median values of SET time (*t*_set_) as a function of programming voltage (*V*_p_) for the Ag/ 3 nm HfO_2_/CE devices with the three different CEs of Pt, TiN, and W. The error bar is the median absolute deviation (MAD) of the experimental data.

Once data were collected from different *V*_p_ and *t*_p_ combinations, the corresponding *t*_set_ values were extracted from the transient current response and plotted semi-logarithmic against *V*_p_ for the three device types with different CEs (Figure 7a–c). In all cases, it can be seen that *t*_set_ exponentially decreases with increasing *V*_p_. The highly non-linear characteristic of *t*_set_ is consistent with previous reports [20,33]. Examining the median of the data in more detail, differences between the devices with various CEs can be obtained, which depend on the voltage regime. All three kinds of devices show marginal differences in their SET kinetics at low voltages (Figure 8). This observation is in agreement with the results from the *I-V* sweeps that fit into this regime of low amplitude and large time span. However, as the voltage increases, at around 0.6 V, the curves for the different CE materials begin to diverge from each other, with Pt showing a steep decrease in SET time followed by TiN and W. 

It has been previously reported that the switching speed in ECM cells is determined by three different rate-limiting factors, which dominate the behavior depending on the selected voltage regime [9,33]. These are (i) nucleation at low voltages, (ii) electron transfer in the medium voltage regime, and (iii) mixed (electron transfer and ion migration) at high voltages. As mentioned earlier, the CE is directly involved in the switching process as it provides a medium for the counter-reaction during the redox process of Ag oxidation and reduction, thus affecting the switching kinetics. Additionally, the interfacial oxide between the HfO_2_-electrolyte and the CE metal should also be taken into account. While reports on the effect of electrolyte thickness on kinetics are scarce, it could be argued that the electric field, which is the driving force for switching, can be reduced for thicker films due to the larger distance between the two conducting electrodes. However, the effect of insulating layer thickness on SET kinetics is very small (usually within the variation range) [31] and thus cannot fully explain the observed differences. Therefore, further considerations are needed, and those will be discussed below. 

Previous works have claimed that differences in SET kinetics for various CEs are due to differences in the electrocatalytic activity of the CE materials [20,21,34]. Inert metals such as Pt, Ir, and Ru have higher catalytic activity compared to non-noble metals [21]. This is because non-noble metals can chemically react and form oxides at the surface. Therefore, for non-noble CE materials, two factors must be taken into account: (1) the CE material itself and its specific catalytic activity towards the redox reaction, and (2) the formation of an interfacial oxide layer at the CE/electrolyte interface. Therefore, the effect of changing the CE material on the SET kinetics of ECM cells should account for the contributions of both factors. 

If devices with rather thick electrolyte layers are switched, the effect of a few nm additional interfacial oxide layer could probably be neglected. However, in this case, where the interfacial oxide layer, in particular, TiO_x_ and WO_x_ for the TiN and W CE, respectively, is almost as thick as the HfO_2_ electrolyte layer, possible influences should be taken into account. Furthermore, regarding only the catalytic activity of the CE, the devices with Pt and TiN CE are expected to show similar kinetics, as seen in the study by Luebben et al. where ECM cells with 10 nm SiO_2_ switching layer were characterized [20]. In contrast to this, the results from thin 3 nm HfO_2_-based ECM cells clearly show a difference in the SET kinetics of Pt and TiN, indicating that additional factors such as the interfacial oxide should be considered. A reasonable assumption for an explanation is that the additional interfacial TiO_x_ layer acts as an additional barrier for Ag^+^ migration and may also affect the counter-reaction at the CE by forming a passivation layer, thus changing the concentration of dissolved Ag ions in the electrolyte compared to a clean HfO_2_/TiN interface. Note that ECM cells with both WO_x_ [35] and TiO_x_ [36] as a switching layer have been reported. 

It should be considered that the thickness of the interfacial metal oxide layer, specifically, TiO_x_ and WO_x_, is influenced by the sequence of the DM device stack, where the deposition of the HfO_2_ film by PE-ALD even enhances the oxidation of the bottom CE material. However, interfacial oxide films can be expected to form wherever a reactive metal oxide and a non-noble metal are adjacent [37]. Therefore, consideration of their impact on the switching speed of ECM cells, and DM devices in particular, could allow further optimization of such devices.

## 4. Conclusions

The influence of CE material on the SET kinetics of Ag/HfO_2_-based DMs was investigated. We found out that non-noble CE materials such as TiN and W form a native oxide either during ambient transfer or during the ALD process of the HfO_2_ electrolyte film. This has been confirmed by the XPS analysis of different stacks with and without the HfO_2_ layer. The analysis of the switching kinetics showed that in the low voltage regime, where nucleation is the rate-limiting factor, the differences between different CE materials are minor. However, at higher voltages, where SET kinetics is limited by electron transfer and ion migration, the SET time differs for the different CE materials. Among the materials studied, Pt showed the fastest SET process, followed by TiN and W. This difference can be explained by (i) the limitation in the counter-reaction, which limits the amount of Ag^+^ available for switching, thus leading to slower SET processes; and (ii) the tendency of the native oxides formed on the surface of the CE material to inhibit the redox reaction at the electrolyte/CE interface by forming a passivation layer. Furthermore, the native oxide can act as an additional diffusion barrier for Ag^+^. These features of non-noble metals are often neglected and, therefore, should be considered in the fabrication and characterization of DM devices. These findings can help in the design of DM cells by choosing the appropriate CE material to optimize the performance and switching speed.

## Data Availability

The data that support the findings of this study are available from the corresponding author upon reasonable request.

## Appendix A

**Table A1:** Sputter parameters for the bottom and top metal layer fabrications.

Parameter	Off-Axis Sputter				On-Axis Sputter	
	Ti Adhesion	Pt BE	TiN BE	W BE	Ag AE	Pt Capping
Sputter type	RF	DC	RF	RF	DC	DC
Sputter power (W)	300	100	100	300	288	359
Ar flow (sccm)	30	30	30	30	60	60
Process pressure (mbar)	0.028	0.055	0.028	0.028	1.2	2.4
Process temperature (°C)	RT					
